# Interleukin-27 Enhances the Potential of Reactive Oxygen Species Generation from Monocyte-derived Macrophages and Dendritic cells by Induction of p47^phox^

**DOI:** 10.1038/srep43441

**Published:** 2017-02-27

**Authors:** Bharatwaj Sowrirajan, Yoshiro Saito, Deepak Poudyal, Qian Chen, Hongyan Sui, Suk See DeRavin, Hiromi Imamichi, Toyotaka Sato, Douglas B. Kuhns, Noriko Noguchi, Harry L. Malech, H. Clifford Lane, Tomozumi Imamichi

**Affiliations:** 1 Laboratory of Human Retrovirology and Immunoinformatics, Leidos Biomedical Research Inc., Frederick National Laboratory for Cancer Research, Frederick, Maryland 21702, USA; 2 Systems Life Sciences laboratory, Department of Medical Life Systems, Faculty of Life and Medical Sciences, Doshisha University, Kyotanabe, Kyoto 610-0394, Japan; 3 Laboratory of Host Defenses, National Institute of Allergy and Infectious Diseases, National Institutes of Health, Bethesda, Maryland 20802, USA; 4 Laboratory of Immunoregulation, National Institute of Allergy and Infectious Diseases, National Institutes of Health, Bethesda, Maryland 20892, USA; 5 Neutrophil Monitoring Laboratory, Leidos Biomedical Research Inc., Frederick National Laboratory for Cancer Research, Frederick, Maryland 21702, USA

## Abstract

Interleukin (IL)-27, a member of the IL-12 cytokine family, plays an important and diverse role in the function of the immune system. We have previously demonstrated that IL-27 is an anti-viral cytokine which inhibits HIV-1, HIV-2, Influenza virus and herpes simplex virus infection, and enhances the potential of reactive oxygen species (ROS) generating activity during differentiation of monocytes to macrophages. In this study, we further investigated the mechanism of the enhanced potential for ROS generation by IL-27. Real time PCR, western blot and knock down assays demonstrate that IL-27 is able to enhance the potential of superoxide production not only during differentiation but also in terminally differentiated-macrophages and immature dendritic cells (iDC) in association with the induction of p47^phox^, a cytosolic component of the ROS producing enzyme, NADPH oxidase, and the increase in amounts of phosphorylated p47^phox^ upon stimulation. We also demonstrate that IL-27 is able to induce extracellular superoxide dismutase during differentiation of monocytes but not in terminal differentiated macrophages. Since ROS plays an important role in a variety of inflammation, our data demonstrate that IL-27 is a potent regulator of ROS induction and may be a novel therapeutic target.

Interleukin (IL)-27, a member of the IL-6/IL-12 cytokine family, is a heterodimer consisting of Epstein-Barr virus-induced gene 3 (an IL-12 p40-related protein) and IL-27 p28 (an IL-12 p35-related protein)^1^. It is mainly produced by dendritic cells and macrophages upon stimulation^2^. Originally identified as a proinflammatory cytokine to induce Th1 responses in T cells^2^,^3^,^4^, IL-27 is also reported to have anti-viral properties including suppression of HIV-1, HIV-2, Hepatitis C virus, Hepatitis B virus and Herpes simplex virus infection^5^. IL-27 binds to the IL-27 receptor, which is a heterodimer composed of IL-27Rα (T-cell cytokine receptor/WSX-1) and gp130, a common receptor chain for the IL-6 cytokine family^1^,^4^, leading to activation of STAT-1 and STAT-3^6^,^7^,^8^. The IL-27 receptor is expressed on T-cells, monocytes, neutrophils, B cells, mast cells, hepatocytes, dendritic cells, and macrophages^9^,^10^,^11^,^12^,^13^,^14^,^15^,^16^,^17^. Accumulating evidence suggests that IL-27 may be an attractive candidate as an immune-therapeutic agent against cancer, allergy, autoimmune diseases, and infectious diseases^5^,^18^,^19^,^20^,^21^.

Reactive oxygen species (ROS), such as hydroxyl radical hydrogen peroxide, and singlet oxygen, are converted from superoxide that is produced by activation of NADPH-oxidase, a membrane-bound enzyme complex that exists in multiple isoforms. ROS generated from NADPH-oxidase plays an important role to protect against infection as well as regulation of signal transduction^22^,^23^. NADPH-oxidase family enzymes include NADPH-oxidase-1 to NADPH-oxidase-5 and DUOX1/2. NADPH-oxidase-2 is expressed on phagocytes and is composed of a total seven subunits: p22^phox^, p40^phox^, p47^phox^, p67^phox^, gp91^phox^, GTPase/Rac1 and GTPase/Rac2. The gp91^phox^ and p22^phox^ subunits are located on the plasma membrane^24^, while the other subunits localize in the cytoplasm. Rac1 and Rac2 are components of the activated NADPH oxidase complex in monocytes/macrophages and neutrophils, respectively^25^,^26^,^27^.

Upon stimulation, p47^phox^ is phosphorylated via a kinase and the phosphorylated p47^phox^ migrates to the plasma membrane where it associates with gp91^phox^ and p22^phox^ to form an active enzyme complex. Increased phosphorylation of p47^phox^ leads to increased activity of NADPH-oxidase and higher levels of ROS. Multiple phosphorylation sites, such as amino acid serine (Ser) at position 303, 304, 328, 358, and 370, in p47^phox^ have been identified as being important sites in assembling the NADPH-oxidase complex^28^. Simultaneous phosphorylation of Ser 303, 304, and 328 unmasks an SH3 domain, resulting in an interaction with p22^phox ^29.

*In vitro* study, monocytes are differentiated into macrophages using cytokines^30^,^31^. GM-CSF and M-CSF-induced macrophages are known as M-1 and M-2 macrophages, respectively. We have previously demonstrated that anti-HIV cytokine, IL-27 promotes macrophages into HIV-resistant macrophages (I-Mac) during differentiation from monocytes without an obvious impact on phagocytosis, chemotaxis, production of pro-inflammatory cytokines such as IL-8, IL-10, TNF-α or MCP-1, and the expression of macrophage differentiation markers such as CD14, CD11B, EMR1 or CD206^32^. Of note, the HIV-resistant I-Mac possess a higher level of potential to produce ROS upon PMA stimulation compared to untreated macrophages and it has been reported that ROS in macrophages is essential for uptake and clearance of apoptotic cells^33^,^34^. In addition, a recent study reported that the inhibition of ROS production blocks differentiation of tumor-associated macrophages and M-CSF-induced monocyte-derived macrophages^35^, thus the enhanced potential of superoxide production in I-Mac may provide a benefit for macrophage function and differentiation. In the current study, we investigated the pathways involved in IL-27 modulation of macrophage function with respect to superoxide production using several types of macrophages and iDC and identified enhanced expression and phosphorylation of p47^phox^ as playing a key role.

## Results

### IL-27 treatment enhances potential ROS production associated with increase in p47^phox^ expression

**Figure 1:**
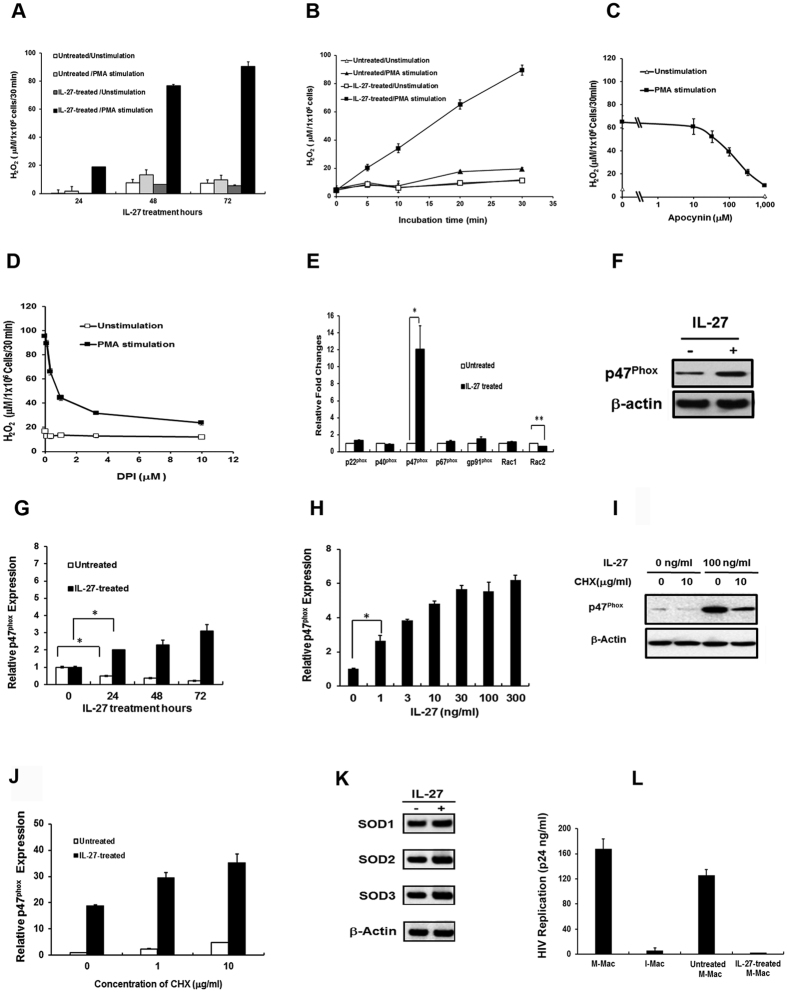
IL-27 treatment enhances NADPH oxidase-2-mediated superoxide production in macrophages. (**a–d**) Superoxide Production was monitored by detecting H_2_O_2_ amounts after stimulation with PMA as described in the Method section. (**a**) M-Mac were treated with 0 or 100 ng/ml of IL-27 for 24, 48 or 72 h followed by stimulation with PMA, (**b**) M-Mac were treated with 100 ng/ml of IL-27 for 48 h and then stimulated with PMA for 5, 10, 20, or 30 min at 37 °C. (**c**,**d**) M-Mac was cultured with 100 ng/ml IL-27 for 48 h, and then pretreated with apocynin or DPI for 30 min before PMA stimulation (**e**,**f**) M-Mac were treated with 100 ng/ml of IL-27 for 48 h. (**e**) the expression of each subunit mRNA of NADPH oxidase-2 was determined by quantitative RT-PCR. Data shown means ± SE of three independent studies. (**f**) The expression of p47^phox^ protein was detected by Western blot. (**g**) M-Mac were treated with 0 or 100 ng/ml of IL-27 for different durations, or (**h**) different doses of IL-27 for 48 h. The relative level of expression of p47^phox^ gene was measured by quantitative RT-PCR. (**i**,**j**) M-Mac were treated with CHX for 30 min at 37 °C and then cultured in the presence of 0 or 100 ng/ml of IL-27 for 48 h. The expression of p47^phox^ protein (**i**) and mRNA (**j**) were analyzed by Western blot and quantitative RT-PCR, respectively. (**k**) M-Mac were incubated with 0 or 100 ng/ml of IL-27 for 48 h and then SOD isotype expression was analyzed by Western blot. (**l**) M-Mac were treated with 0 or 100 ng/ml of IL-27 for 48 h and then infected with HIV. The HIV infected cells were cultured for 14 days and then HIV replication was monitored by p24 antigen capture kit. Data shown represent means ± SDs of triplicate samples of two independent experiments. *P < 0.01, **P < 0.05 In Western blot analysis, anti-b-actin antibody was used to determine an internal control.

In our previous work, we demonstrated that macrophages differentiated from monocytes in the presence of IL-27 (IL-27-induced macrophages: I-Mac) resist infection of HIV-1, HIV-2, Influenza virus, and Herpes simplex virus, and produced 6-fold higher levels of ROS production upon PMA stimulation than M-CSF-induced macrophages (M-Mac) upon stimulation^32^. The data indicated that IL-27 enhances potential of superoxide production during differentiation, however, the mechanism underlying this observation has, thus far, remained unclear. To explore the mechanisms beneath the ROS inducing activity of IL-27, we first examined the effects of IL-27 directly on terminally differentiated M-Mac. M-Mac were treated with IL-27 for 0, 24, 48 and 72 h and then stimulated with PMA for 30 min prior to measurement of superoxide as reflected by hydrogen peroxide (H_2_O_2_) production. PMA stimulation of IL-27-treated M-Mac enhanced ROS production in a time dependent manner (Fig. 1a). Furthermore, IL-27-treated M-Mac continued to produce ROS for at least 30 min (Fig. 1b). This indicates that IL-27 is able to enhance the potential for ROS production by even fully differentiated macrophages.

The induction of ROS from phagocytes is mediated by NADPH-oxidase-2. To confirm whether or not the enhanced ROS induction from IL-27-treated M-Mac is mediated by NADPH-oxidase-2, we evaluated the impact of apocynin and diphenylene iodonium (DPI) on the superoxide production. Apocynin is a specific inhibitor of NADPH oxidase-2^36^. DPI inhibits NADPH oxidase-2 by removing an electron from the reduced NADPH-oxidase enzyme^37^. As shown in Fig. 1c,d, although trypan blue exclusion assay demonstrated any significant cell toxicity (data not shown), the production of ROS was dose-dependently suppressed by either apocynin or DPI implicating NADPH oxidase-2 as a critical mediator of this activity.

NADPH oxidase-2 is composed of p22^phox^, p40^phox^, p47^phox^, p67^phox^, gp91^phox^, and Rac1/2 subunits. To determine if IL-27-mediated ROS enhancement was due to an increase in some of the subunits of NADPH oxidase-2, we examined the relative levels of gene expression of each subunit of NADPH oxidase-2 in IL-27-treated and untreated M-Mac. The expression of the p47^phox^ gene was increased approximately 12-fold (P < 0.01) and Rac2 gene was downregulated by near 50% (p < 0.05) in IL-27-treated M-Mac compared to untreated M-Mac, however, the expression of other subunits was not significantly changed (Fig. 1e). The increase in p47^phox^ gene expression was associated with an increase in p47^phox^ protein expression (Fig. 1f and Supplementary Fig. S1) by 2.01 ± 0.11 (n = 5) fold. To further characterize the gene induction by IL-27, time course and dose response analyses were performed. The endogenous expression of p47^phox^ was decreased in untreated cells after 24 h incubation (p < 0.01), while the expression level of p47^phox^ in IL-27-treated cells was increased, rather than maintained, in a time dependent manner (Fig. 1g) (p < 0.01). The induction was saturated at more than 30 ng/ml of IL-27 at 48 h incubation (Fig. 1h). In our previous work, we have used 100 ng/ml of IL-27 to stimulate macrophages or differentiate monocytes into macrophages^13^,^32^,^38^,^39^, thus in this study, we used the same concentration of IL-27 in downstream experiments. Translational inhibitor Cycloheximide (CHX) was used to define whether the increase in p47^phox^ protein is associated with de novo synthesis or protein stability. CHX inhibited the p47^phox^ protein expression dose dependent manner (Fig. 1i) with the accumulation of p47^phox^ mRNA (Fig. 1j). Taken together, these data suggesting that IL-27-mediated increase in p47^phox^ is associated with induction of p47^phox^ transcripts rather than either stabilization of the p47^phox^ mRNA or p47^phox^ protein. In this study, superoxide produced by NADPH oxidase-2 was monitored by detecting produced H_2_O_2_. The superoxide is catalyzed into H_2_O_2_ by super oxide dismutase (SOD)^40^, therefore, it was considered that the augmented H_2_O_2_ production may be associated with the increase in SOD expression in IL-27-treated M-Mac. To define the role of IL-27 on the expression of SOD, western blot analysis was performed. SOD exists in three isoforms in mammals: the cytoplasmic Cu/Zn-SOD (SOD1), the mitochondrial Mn-SOD (SOD2), and the extracellular Cu/Zn-SOD (SOD3)^41^, thus, the expression level of the three isoforms in IL-27-treated M-Mac were compared to that in untreated M-Mac. The Western blot analysis revealed that none of the isoforms were significantly changed in IL-27-treated M-Mac as compared to untreated M-Mac (Fig. 1k and Supplementary Fig. S2).

We have previously demonstrated that IL-27 induced macrophages (I-Mac) resist HIV infection^32^. To elucidate whether or not the IL-27-treated M-Mac possess resistance to HIV infection as I-Mac does, anti-HIV effect was evaluated. IL-27-treated M-Mac or untreated M-Mac were infected with HIV-1 and the amount of HIV-1 p24 released into the supernatant was assessed. HIV-1 infection of I-Mac was also analyzed as a control. As shown in Fig. 1l, the IL-27-treated cells demonstrated HIV resistance as I-Mac, indicating that IL-27 is able to induce HIV-resistance property in terminally differentiated macrophages.

**Figure 2:**
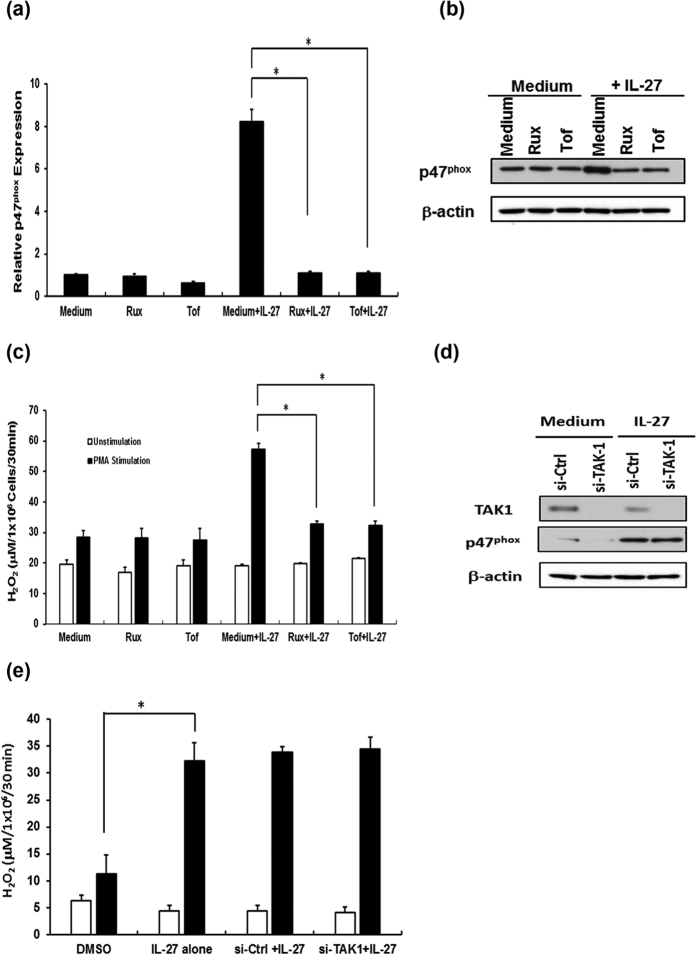
JAK inhibitors suppress the IL-27 induction of p47^phox^ expression and PMA-induced superoxide production. M-Mac were treated with 5 μM of Tofacitinib (Tof) or 1 μM of Ruxolitinib (Rux) for 1 h at 37 °C, and then cells were cultured for 48 h in the presence or absence of 100 ng/ml of IL-27. (**a**) Total cellular RNA was extracted from the cells and then p47^phox^ mRNA expression was detected by real time PCR as described in the experimental procedures. Data show representative means ± SDs of 3 independent experiments. (**b**) Total cell lysate was prepared with RIPA buffer and then western blotting was performed using anti-p47^phox^ antibody. Anti-β Actin antibody was used as a loading control. (**c**) PMA-induced superoxide production from the inhibitor-treated cells were measured as described in the experimental procedure. Data show means ± SDs and are representative of 2 independent experiments. (**d**) M-Mac were transfected with 100 pmol si-RNA against TAK-1 (si-TAK) or control si-RNA (si-Ctrl) as described in the experimental procedure, and then cultured with or without 100 ng/ml of IL-27 for 48 h. The expression of TAK-1 and p47^phox^ were analyzed by western blotting. (**e**) si-TAK-1 or si-Ctrl-transfected cells were stimulated with or without 100 ng/ml PMA for 30 min and then superoxide production was monitored as described in the experimental procedure. Data show means ± SDs and are representative of 3 independent experiments. *P < 0.01.

Having established that IL-27 has an effect on p47^phox^ gene expression, we next sought to determine the signaling pathway involved in this modulation. IL-27 is known to trigger JAK1 and JAK2 activation followed by STAT-1, -3 and -5 signaling pathway^2^. In our previous work, we demonstrated that IL-27 induces TGF-β activated kinase-1 (TAK-1) signaling pathway in M-Mac^32^. To determine which pathway is involved in the p47^phox^ expression and superoxide production, we performed inhibition assays. Prior to the IL-27 treatment, M-Mac were pre-incubated with JAK inhibitors and then p47^phox^ expression and superoxide production were analyzed. 1 μM of Ruxolitinib (JAK1/2 inhibitor) and 5 μM of Tofacitinib (JAK1/3 inhibitor) suppressed the IL-27-mediated STAT-1, -3 and -5 activation (Supplementary Fig. S3) without any significant change in cell viability (data not shown). Using this concentration of inhibitors, p47^phox^ expression and superoxide production were analyzed. Both JAK inhibitors abolished the IL-27-mediated induction of p47^phox^ gene activation, protein production (Fig. 2a,b) and PMA-stimulated superoxide production (Fig. 2c). To address a role of TAK-1 pathway in the p47^phox^ induction and superoxide production, M-Mac were treated with a small interfering RNA (siRNA) against TAK-1 (si-TAK-1) or NG25, a TAK-1 specific inhibitor. Although 100 pmol of si-TAk-1 knocked down TAK-1 expression by 77% (Fig. 2d), the knockdown had no impact on either the induction of p47^phox^ expression or superoxide production (Fig. 2d,e), even TAK-1 expression was knocked down, superoxide production was induced by ~6-fold compared to unstimulated cells as si-Ctrl treated cell does. 100 nM of NG25 treatment abrogated TAK-1 mediated activation of p38 MAPK (Supplementary Fig. S4a) without any significant changes in IL-27-mediated p47^phox^ induction and superoxide production (Supplementary Fig. S4b,c). Taken together, these data indicate that the JAK/STAT, but not TAK-1 pathway plays a pivotal role in IL-27-mediated p47^phox^ induction.

### Direct impact of knockdown or overexpression of p47^phox^ on Superoxide production

**Figure 3:**
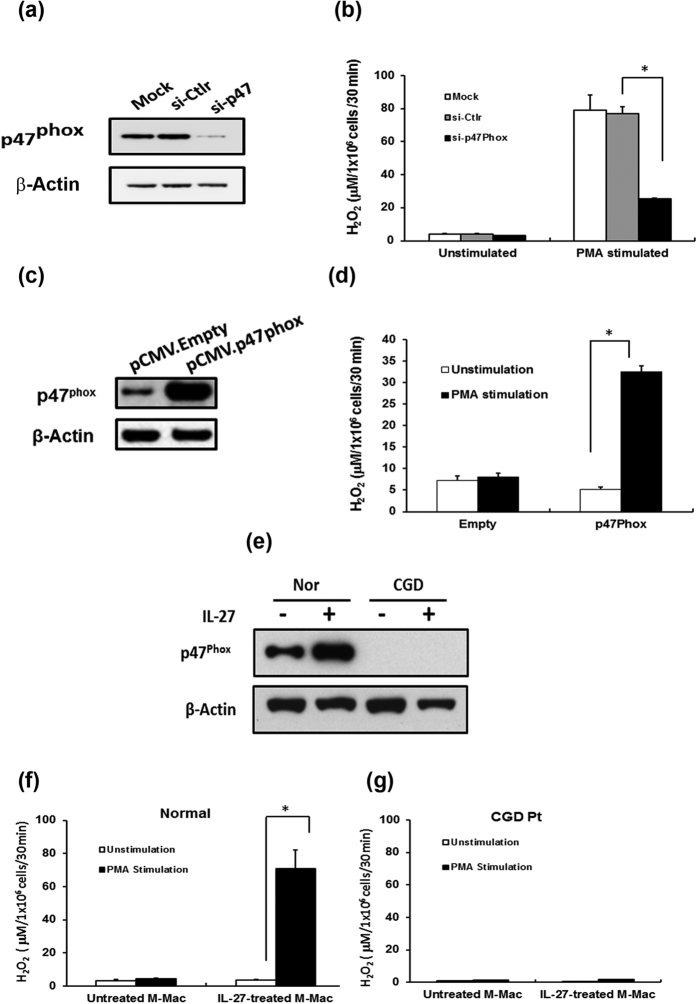
IL-27 increases the expression of p47^phox^ in macrophages. **(a**) M-Mac were transfected with either control si-RNA (si-Ctlr) or si-RNA targeting p47^phox^ (si-p47) followed by treatment with or without 100 ng/ml IL-27 for 48 h at 37 °C. As a control, un-transfected cells (Mock) were treated with IL-27. 48 h after transfection, whole cell lysates were prepared and western blotting was performed using anti-p47^phox^ or anti-β-actin antibodies. (**b**) si-Ctlr, si-p47 or Mock-transfected cells were treated with IL-27 for 48 h at 37 °C and superoxide production was analyzed by measuring H_2_O_2_ within culture supernatants. Data shown represent means ± SD of triplicate samples. (**c**) Macrophages were transfected with an empty or a p47^phox^ expression vector for 6 h, and then the expression levels of p47^phox^ were determined by Western blot. (**d**) H_2_O_2_ induction by PMA stimulation was detected by ROS assays following transfection of either the empty or p47^phox^ expression vector into macrophages. Data shown represent means ± SD of triplicate samples. (**e**,**f**) Monocytes from a healthy control or a CGD patient lacking the expression of p47^phox^ were differentiated into macrophages. Cells were then incubated with or without IL-27 for 48 h at 37 °C. The resulting cells were then stimulated with or without 100 ng/ml PMA for 30 min and ROS activity was measured by detection of H_2_O_2_ in culture supernatants. Data shown represent means ± SDs of triplicate samples from three independent studies. *P < 0.01.

To further examine the direct role of p47^phox^ in the IL-27 associated increase in generation of ROS following PMA stimulation, p47^phox^ was knocked down using si-RNA against p47^phox^ (si-p47). Cells treated with si-p47 had significantly lower expression of p47^phox^ (Fig. 3a) (10% of expression of p47^phox^ as compared to control), and significantly decreased the amounts of PMA-induced ROS production (p < 0.01) (Fig. 3b). To determine the impact of overexpression of p47^phox^, a p47^phox^ expression vector was constructed and transfected into M-Mac followed by measuring PMA-induced superoxide production. The expression vector enhanced p47^phox^ expression by 4-fold (Fig. 3c), and PMA-induced superoxide production was enhanced by 6-fold (Fig. 3d) as compared to un-transfected cells (p < 0.01). Thus, the increased expression of p47^phox^ even in the absence of IL-27 leads to an enhancement in ROS production from macrophages.

### IL-27 treatment of macrophages from a p47^phox-/-^ CGD patient fails to induce reactive oxygen species

One form of chronic granulomatous disease (CGD) is caused by missense, nonsense, frame shift, splice, or deletion mutations in p47^phox^, resulting in impaired production of ROS^42^. To further define the role of IL-27 in enhancing the potential for ROS production, superoxide production was analyzed from IL-27 stimulated macrophages derived from a p47^phox−/−^ patient. This patient’s cells lack the expression of p47^phox^ as compared to a healthy donor (Supplementary Fig. S5). Other NADPH oxidase-2 subunits (gp91^phox^, p67^phox^, p40^phox^, p20^phox^ and Rac1) were expressed to comparatively similar levels as a healthy donor (Supplementary Fig. S5). IL-27 treatment of p47^phox−/−^ macrophages failed to induce either p47^phox^ expression (Fig. 3e) or ROS production (Fig. 3f,g). These data strongly suggest that p47^phox^ is a critical mediator of the effect of IL-27 on PMA-induced superoxide production.

### IL-27-enhanced PMA-induced phosphorylation of p47^phox^ is required for Superoxide production

**Figure 4:**
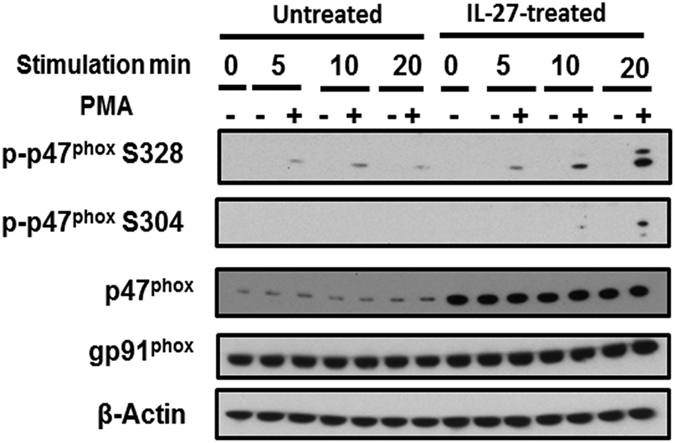
IL-27 enhances phosphorylation of p47^phox^ in macrophages. IL-27 treated and untreated macrophages were stimulated with 100 ng/ml PMA for 20 min at 37 °C and then whole cell lysates were prepared in the presence of a phosphatase inhibitor and proteinase inhibitor. Western blot was performed with anti-phosphorylated p47^phox^, anti-p47^phox^, anti-gp91^phox^, and anti-β-actin was used for detecting a loading control.

In addition to increasing total levels of p47^phox^, it is possible that IL-27 might modulate p47^phox^ activity by influencing its phosphorylation. An increase in p47^phox^ phosphorylation is associated with enhanced superoxide production^28^. Therefore, we compared the phosphorylation of p47^phox^ between IL-27-treated and untreated macrophages. Both macrophages were stimulated with PMA for 20 min and whole cell lysates were prepared after 0, 5, 10 and 20 min stimulation and then phosphorylation of p47^phox^ was analyzed by western blot. Before PMA stimulation, phosphorylation at S328 or S304 on p47^phox^ was not detected in both untreated and IL-27-treated M-Mac. After 5 min of PMA-stimulation, phosphorylation at S328 was initially induced in both cell types, and then 10 min after the stimulation, the amount of phosphorylation in untreated cells was reduced, while the amount in the IL-27-treated cells was increased until 20 min stimulation. In addition, the phosphorylation at S304 was increased in the IL-27-treated macrophages (Fig. 4).

### IL-27-induced macrophages promotes p47^phox^ expression and enhance potential superoxide production

**Figure 5:**
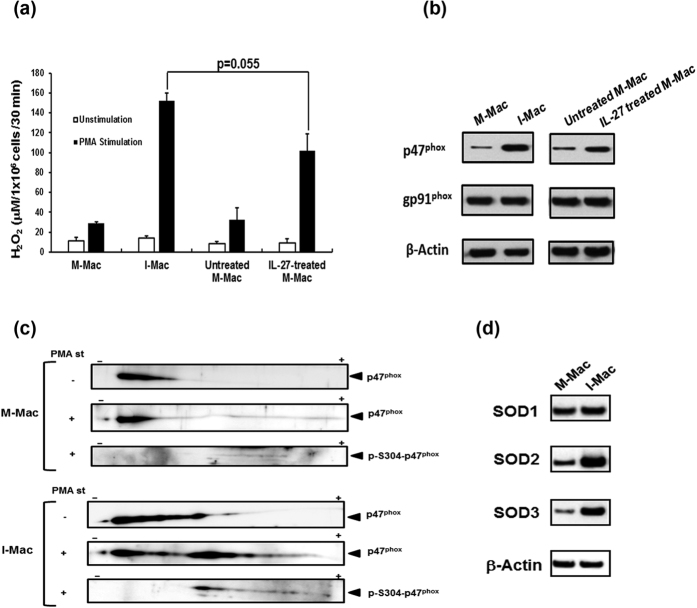
The expression of p47^phox^ is increased in IL-27-induced macrophages (I-Mac). Monocytes were differentiated into macrophages in the absence (M-Mac) or presence of IL-27 (I-Mac). After differentiation, cells were incubated with or without IL-27. (**a**) M-mac, I-Mac, untreated M-Mac and IL-27-treated M-Mac were stimulated with or without 100 ng/ml PMA for 30 min at 37 °C and then ROS activity was measured by detection of secreted H_2_O_2_. Data shown represent means ± SD of triplicate samples. (**b**) The expression levels of p47^phox^ were determined by western blotting for M-Mac, I-Mac, untreated M-Mac and IL-27-treated M-Mac. (**c**) M-Mac and I-Mac were stimulated with or without PMA for 30 min, and then cell lysates were subjected to 2D gel electrophoresis. Expression of p47^phox^ was detected by anti-p47^phox^ and anti-phosphorylated S304 p47^phox^ antibodies. (**d**) Whole cell lysates of M-Mac and I-Mac were prepared using RIPA buffer. Western blot analyses were performed using anti-SOD1, SOD2, SOD3 or anti-β-actin antibodies. The intensity of the band was analyzed by NIH Image J and normalized each SOD intensity with β-Actin.

In our previous work, we demonstrated that macrophages differentiated in the presence of IL-27 (I-Mac) resist HIV and other viral infections^32^. In addition, the potential of superoxide production was enhanced in the cells^32^. To precisely compare the potential of superoxide production among M-Mac, I-Mac, untreated M-Mac and IL-27-treated M-Mac, these cell types were generated from the same lot of monocytes, and then p47^phox^ protein expression and superoxide production were compared. I-Mac and IL-27-treated M-Mac produced 5- and 2-fold higher levels of ROS as compared to M-Mac and untreated M-Mac, respectively (Fig. 5a). In addition, protein amounts of p47^phox^ was enhanced in I-Mac and IL-27-treated M-Mac by 6- and 2-fold, respectively (Fig. 5b). To illustrate phosphorylation level of p47^phox^, PMA-stimulated and unstimulated cells were lysed and 2D Western blot analysis was performed using the lysate. Cell lysate from PMA-stimulated I-Mac resulted in smearing p47^phox^ band shifting to (+) side on the gel (Fig. 5c). This result indicated that PMA-stimulated cells may contain highly phosphorylated p47^phox^ forms. To define whether the smear band is associated with an increase in phosphorylation, 2D-western blotting was performed using anti-phosphorylated S304 p47^phox^ antibody. The p47^phox^ band was detected only in a cell lysate from PMA-stimulated I-Mac. To further demonstrate that the migration toward (+) side is caused by phosphorylation rather than some other modification of p47^phox^, a cell lysate from the PMA-stimulated I-Mac were treated with or without Lambda protein phosphatase *in vitro,* and then were subjected to 1D and 2D western blot. The phosphatase treatment decreased the level of phosphorylated p47^phox^ in the 1D western blotting (supplementary Fig. S6a) and suppressed the shift of the p47^phox^ band to (+) side (Supplementary Fig. S6b). Taken together, these results illustrate that PMA–stimulated I-Mac express higher levels of phosphorylated p47^phox^ than M-Mac. As shown in Fig. 1k, IL-27 has no impact on the expression of the three isoforms of SOD in terminally differentiated macrophages. To define the role of IL-27 on SOD expression during monocytes differentiation, the expression was compared between M-Mac and I-Mac by Western blot. Although SOD1 expression was unchanged, of interest, the protein expression level of SOD2 and SOD3 in I-Mac were increased by 2.5 ± 0.6 (n = 4, p < 0.01) and 4.7 ± 2.3 (n = 4, p < 0.01), respectively (Fig. 5d and Supplementary Fig. S7) compared to M-Mac.

### IL-27 treatment enhances potential ROS production in iDC and other monocytes-derived macrophages

**Figure 6:**
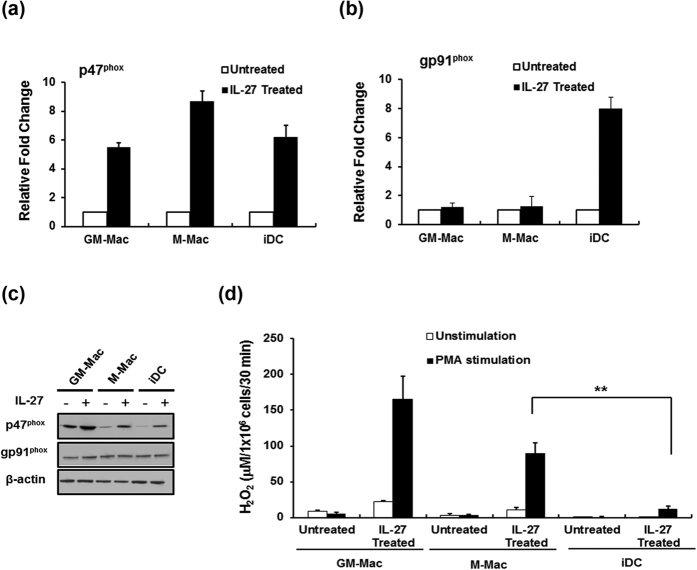
IL-27 enhances superoxide production in GM-CSF-induced macrophages and iDC. (**a**) Monocytes were differentiated in the presence of GM-CSF and IL-4 into iDCs, GM-CSF alone into GM-Mac, or M-CSF alone into M-Mac from the same lot of monocytes. Differentiated cells were subsequently cultured for 48 h at 37 °C either in the absence or presence of 100 ng/ml IL-27. Using gene specific probes, mRNA expression levels of p47^phox^ and gp91^phox^ were quantified by RT-PCR. Values were normalized to GAPDH levels in untreated cells. Data shown represent means ± SE of three independent studies. (**b**) Whole cell lysates from iDCs, GM-Mac, and M-Mac with or without stimulation by IL-27 were analyzed by western blotting for p47^phox^, gp91^phox^, and β-actin expression as an internal control. (**c**) GM-Mac, M-Mac and iDC were cultured in the presence or absence of IL-27 and then treated with or without 100 ng/ml of PMA for 30 minutes. ROS activity was measured in the culture supernatants. Data shown represent means ± SE of three independent studies. **P < 0.05.

In addition to M-CSF induced macrophages (M-Mac), the NADPH-oxidase-2 family of enzymes is also found in GM-CSF induced macrophages (GM-Mac) and monocyte derived dendritic cells (iDC)^43^,^44^. M-Mac, GM-Mac and iDC were differentiated from a same lot of monocytes and then treated with IL-27 to determine each cell type’s ability to induce p47^phox^ expression and subsequent superoxide production in response to PMA. Following their respective differentiation, M-Mac, GM-Mac and iDC were exposed to IL-27 for 48 h prior to analysis for p47^phox^ and gp91^phox^ induction and ROS production. M-Mac, GM-Mac and iDC all revealed increased amounts of p47^phox^ mRNA following IL-27 exposure (Fig. 6a). The mRNA level of gp91^phox^ also increased in only iDC. Induction of the p47^phox^ mRNA in GM-Mac and iDC corresponded to an increase in p47^phox^ protein expression (Fig. 6c). IL-27 treatment of GM-Mac and iDC revealed a 1.7 and 10.1-fold increase in p47^phox^ protein expression, respectively (Fig. 6b). A 1.5-fold increase in gp91^phox^ protein level was observed in iDC treated with IL-27 as compared to untreated iDC. The relative potential ROS production of GM-Mac, M-Mac and iDC were also measured following IL-27. Exposure of cells to PMA for 30 min following IL-27 treatment of M-Mac, GM-Mac and iDCs induced ROS production by 60, 165 and 12-fold compared to untreated cells, respectively (Fig. 6c). These findings show that IL-27 is able to increase p47^phox^ expression in differentiated GM-Mac and iDC as well as M-Mac and that this increase is associated with an enhanced potential for superoxide production.

## Discussion

**Figure 7:**
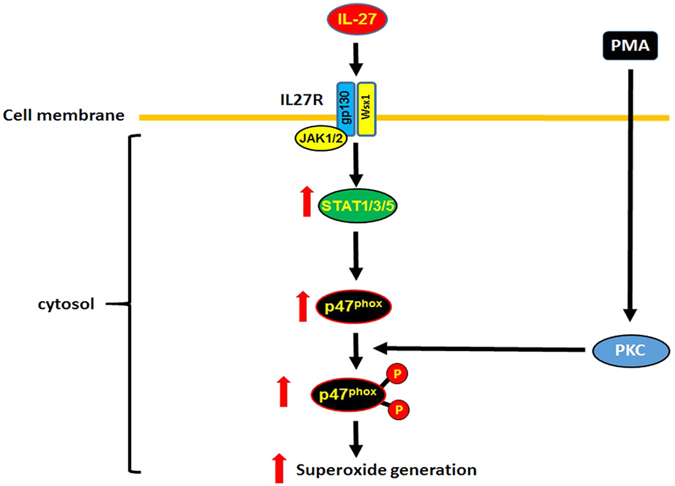
A schematic model of IL-27 effect on superoxide generation. IL-27 binds to IL-27 receptor composing of gp130 and WSX1^4^. The binding induces JAK/STAT activation followed by enhancement of p47^phox^ expression in macrophages. PMA stimulation activates PKC and augments phosphorylation of p47^phox^, subsequently the highly phosphorylated p47^phox^ is involved in increase in the superoxide generation.

IL-27 has emerged as an important immunomodulatory cytokine playing pivotal roles in both innate and adaptive immunity^5^. Our previous studies reported that IL-27 differentiates monocytes into HIV-resistant macrophages (IL-27-induced Mac: I-Mac) and enhanced a potential activity of superoxide production in the cells upon PMA stimulation^32^, suggesting that IL-27 enhances a potential of ROS inducing activity during differentiation. In this study, we have further demonstrated that IL-27 is able to increase the potential for superoxide production even in terminally differentiated macrophages, in addition, IL-27 treatment leads to HIV resistance in the terminally differentiated M-CSF-induced macrophages, indicating that IL-27 is able to modulate macrophage functions during and after differentiation. Furthermore, this increase in the potential for superoxide production is associated with an induction of p47^phox^, a component of the NADPH-oxidase-2 complex. This augmentation is mediated via JAK/STAT pathway and increase in phosphorylation of p47^phox^ (Fig. 7). The p47^phox^ was augmented in all tested macrophages subtypes (M-mac and GM-mac). We observed an increase in p47^phox^ at the mRNA and protein levels, silencing of p47^phox^ in IL-27-treated M-Mac suppressed superoxide production, and IL-27 failed to induce ROS activity from p47^phox−/−^ macrophages derived from a CGD patient with a genetic defect in p47^phox^. Of note, transfection of a p47^phox^ expression plasmid in M-Mac increased the p47^phox^ expression by 4-fold, but it only enhanced ROS production by 2~3-fold, since IL-27 treatment had no impact on SODs expression, the data suggest that other factors in addition to p47^phox^ induction may be involved in the enhancement of potential of superoxide production in IL-27-treated cells. Despite the fact that IL-27 increases the p47^phox^ expression, without a stimulation, IL-27-treated and untreated cells had indistinguishable levels of spontaneous superoxide production. Therefore, the increase in potential of superoxide production may not directly influence macrophage differentiation and function without stimulation. In this study, the potential for superoxide production by NADPH oxidase activity was monitored by detecting H_2_O_2_ production. The produced superoxide is catalyzed to H_2_O_2_ by SOD^41^, therefore, it was considered that the increase in H_2_O_2_ production may be associated with increase in SOD expression. Although IL-27-treated M-Mac demonstrated little if any change on the expression of all three types of SOD isoforms, of note, in I-Mac, the expression of SOD2 and SOD3, but not SOD1, were significantly increased compared to that in M-Mac. ROS production from I-Mac was relatively higher than that from M-Mac. It is known that SOD2 plays a key role on mitochondrial NADPH oxidase activity^41^, thus it is possible that the increase in the SOD2 expression in I-Mac contributes in the enhanced ROS production. SOD3 was originally discovered as a secreted, extracellular protein^45^, however, current studies demonstrate that SOD3 is expressed on the cell surface^46^,^47^,^48^. Thus the induced SOD3 may also contribute to the enhanced ROS production by I-Mac. Additionally, it was reported that SOD3 on macrophage is associated with strong bacterial killing by phagocytosis^47^. Thus, IL-27-indcued macrophages (I-Mac) may also possess an increased anti-bacterial activity. Further study may define the mechanism of SOD3 induction in I-Mac and delineate insight of a role of SOD3 in virus infection in macrophages.

Real time PCR and western blotting confirmed that IL-27 induced p47^phox^ during and/or after differentiation of monocytes to macrophages and iDC. This increase corresponded to enhancement of superoxide production upon stimulation in all cell types. These findings suggest that IL-27 may be capable of enhancing ROS production in myeloid lineage including microglia cells^49^. IL-27 treatment enhanced the expression of gp91^phox^ mRNA in only iDC; however, this induction did not correspond with protein induction. The potential activity of superoxide production was compared among M-Mac, GM-Mac and iDC. Despite the fact that IL-27 treatment induced p47^phox^ in all these cells types, the induction level of ROS from iDCs was 1/10~1/20 compared to the level from M-Mac and GM-Macs. To further delineate the ability of IL-27 on p47^phox^ induction in different types of macrophages, p47^phox^ expression was analyzed on macrophages differentiated in the presence of GM-CSF and IL-27 (I-GM-Mac). To precisely compare the IL-27 effect on different macrophages, monocytes from the same lot of donors were differentiated into M-Mac, I-Mac, GM-Mac and I-GM-Mac. Western blot analysis revealed that the expression of p47^phox^ protein was enhanced by 5.2 + 1.9-fold (p < 0.01, n = 4) in I-Mac and by 3.9 + 1.4-fold (p = 0.051, n = 4) in I-GM-Mac (Supplementary Fig. S8), suggesting that IL-27 is able to induce p47^phox^ during I-GM-Mac differentiation. Further study is needed to define the mechanism underlying these differences among these cell types.

To define whether or not IL-27 induced p47^phox^ expression is caused by the induction of transcripts or the stabilization of p47^phox^ mRNA, time course and dose response assays were performed. In untreated cells, the endogenous expression of level of p47^phox^ mRNA was time-dependently decreased, whereas, in IL-27-treated cells, the expression level was increased by time dependent and a dose dependent manner and the induction was inhibited by JAK but not TAK1 inhibitors. A translational inhibitor, CHX, treatment decreased the p47^phox^ protein amount along with accumulation of p47^phox^ mRNA. Taken together, IL-27 induces the p47^phox^ mRNA expression rather stabilize either mRNA or protein. Further study may need to elucidate a molecular mechanism of the IL-27-mediated p47^phox^ induction. IL-27 belongs to the IL-6 family cytokine based on gp130 usage. The IL-6 family is composed of IL-6, IL-11, IL-27, IL-31, IL-35, leukemia inhibitory factor (LIF), Oncostatin M (OSM), and ciliary neurotrophic factor (CNTF), and induces activation of STAT-1, 3, and 5^50^,^51^,^52^. To determine whether or not those cytokines rather than IL-27 also increase in p47^phox^ expression, we incubated macrophages with a variety of cytokines and then analyzed p47^phox^ gene expression. IL-27 was the only clear inducer of p47^phox^ in this family (Supplementary Fig. S9), suggesting that IL-27 may differentially regulate signal transduction via gp130.

In our previous study (29), we demonstrated that IL-27 induces HIV resistance in macrophages with an enhancement of potential of superoxide production. In this study we demonstrated IL-27 is able to enhance the potential in not only M-CSF-induced macrophages, but also GM-CSF-induced macrophages and dendritic cells, therefore, further study of role of IL-27 on the differentiated macrophages and dendritic cell may shed light on a new physiological function of IL-27. ROS plays a significant role in a variety of inflammatory diseases and it is noted that ROS in macrophages is essential for uptake and clearance of apoptotic cells^33^,^34^ and ROS production is an essential factor for differentiation of macrophages^35^, thus enhanced potential of superoxide production by IL-27 may provide a benefit for macrophage function and differentiation. IL-27, a potent regulator of ROS production in myeloid lineage cells, may be a novel therapeutic target for one or more of these conditions.

## Methods

Approval for these studies including all sample materials was granted by the National Institute of Allergy and Infectious Diseases Institutional Review Board and participants were informed written consent prior to blood being drawn. Participants were informed written consent prior to blood being drawn. All experimental procedures in these studies were approved by the National Cancer Institute at Frederick and performed in accordance with the relevant guidelines and regulations.

### Cells and reagents

CD14^+^ monocytes were purified from PBMCs of healthy donors using CD14 MicroBeads (Miltenyi Biotec) according to the manufacturer’s instructions as previously described^38^. Approval for these studies was granted by the National Institute of Allergy and Infectious Diseases Institutional Review Board. Participants were informed written consent prior to blood being drawn. To generate M-CSF induced macrophages (M-Mac) or GM-CSF-induced macrophages (GM-Mac), the isolated CD14^+^ monocytes were cultured for 7 days in the presence of 25 ng/ml M-CSF (R&D systems) or 50 ng/ml of GM-CSF (R&D systems) in macrophage serum-free medium (Thermo Fisher Scientific) supplemented with 10 mM HEPES and 5 μg/ml of Gentamycin. IL-27-induced macrophages (I-Mac) were generated from the CD14^+^ monocytes by culturing for 7 days in the presence of 25 ng/ml of M-CSF with 100 ng/ml of IL-27 (R&D systems)^32^. M-Mac and I-Mac were then maintained in D-10 medium [D-MEM (Thermo Fisher Scientific) containing 10% FBS (HyClone Laboratories), 25 mM HEPES, and 5 μg/ml Gentamicin. To induce immature dendritic cells (iDC), CD14^+^ monocytes were cultured at 0.5 × 10^6^ cells/ml for 7 days in the presence of 50 ng/ml GM-CSF, 50 ng/ml of IL-4 (R&D systems) in RPMI-1640 (Thermo Fisher Scientific) with 10% FBS, 10 mM HEPES, and 5 μg/ml of Gentamycin (G4 media)^13^. Cell viability was determined by trypan blue exclusion assay using a final 0.2% trypan blue solution (Thermo Fisher Scientific). JAK inhibitors, Ruxoliotinib and Tofacitinib^53^ were obtained from Selleckchem. A translational inhibitor, cycloheximide was obtained from Sigma-Aldrich. Anti-SOD1 antibody (#2770), anti-SOD2 antibody (#13194), anti-p47^phox^ antibody (#4312) and anti-phosphorylated p38MAPK antibody (#4511) were obtained from Cell Signaling. Anti-phosphorylated p47^phox^ antibodies (ab63554 and ab111855) and anti-gp91^phox^ antibody (ab31092), and anti-p40^phox^ antibody (ab2244) were purchased from Abcam. Anti-Rac1 antibody (# 05-384) and Anti-β-actin antibody (AC-15) was purchased from EMD Millipore and SantCruz, respectively. Si-RNA against TAK-1 (#s13788) was obtained from Thermo Fisher Scientific.

### Superoxide Production Assay

Superoxide producing activity were determined by measuring levels of hydrogen peroxide (H_2_O_2_) using the Amplex Red Hydrogen Peroxide/Peroxidase Assay Kit (Thermo Fisher Scientific). Briefly, macrophages or dendritic cells were seeded in 96 well plates (5 × 10^4^ cells/well for macrophages and 1 × 10^5^ cells/well for iDC) in D-10 or G4 media, respectively, and incubated at 37 °C overnight in 5% CO_2_. Cells were then washed 3X with warmed Krebs–Ringer phosphate buffer (145 mM NaCl, 5.7 mM sodium phosphate, 4.86 mM KCl, 0.54 mM CaCl_2_, and 1.22 mM MgCl_2_) supplemented with 5.5 mM Glucose and then incubated in the buffer for 30 min at 37 °C before stimulation. For stimulating and detecting ROS production, 50 μL of prewarmed 2xAmplex Red reagent (Thermo Fisher Scientific) containing 200 ng/mL PMA (Sigma-Aldrich) was added to each well and the plates incubated for 30 min at 37 °C. As a control, some wells did not receive PMA. The induced H_2_O_2_ was measured at 560 nm in a 96 well plate reader (PerkinElmer). To quantitate the produced H_2_O_2_, 20 mM stable H_2_O_2_ (Thermo Fisher Scientific) was used for a standard.

### HIV replication assay

Macrophages were infected with HIV as previously described^32^. Briefly, M-Mac, I-Mac, or IL-27 treated cells 5 × 10^6^ cells were incubated with 5000 TCID_50_ HIV-1_BAL_ (Advanced Biotechnologies, Inc) for 2 h. The HIV infected cells were cultured for 14 days in D-10 media in 96 well plates. Half culture supernatants were replaced with fresh media every 3 or 4 days. HIV replication activity was monitored using an HIV p24 antigen capture kit (Perkin-Elmer).

### Construction of p47^phox^ expression plasmid

The p47^phox^ expression plasmid (pCMVp47Phox) was conducted as follows: p47phox cDNA was synthesized from 5 μg of total cellular RNA derived from macrophages using the Superscript First Stand Synthesis System for RT-PCR (Thermo Fisher Scientific). PCR amplification of the cDNA using the PCR primer pair: 5′-AGC CGC CAT GGG GGA CAC CTT CAT C-3′ and 5′-GGT ACC CTA GAC GGC AGA CGC CAG CTT CCG CTT G-3′ with the Expand High fidelity PCR system (Roche Molecular Diagnostics). The PCR product was ligated into pCR2.1 and confirmatory DNA sequencing was performed using BigDye 3 with ABI Prism 3130X Genetic Analyzer. The p47phox gene intended gene was then subcloned into pCMV5a (Sigma-Aldrich) to generate pCMVp47Phox.

### Quantitative RT-PCR

Macrophages or iDCs were washed with cold PBS (Quality Biology) and RNA was isolated from the cells using the RNeasy Isolation kit (Qiagen). Total cDNA was then synthesized using Taqman reverse transcription reagents (Thermo Fisher Scientific) with random hexamer priming. Expression levels of the genes of interest were measured by semi-quantitative RT-PCR by a CFX96 Real-Time system (BioRad). The level of gene expression were normalized to GAPDH. Probes specific for each subunit of NADPH oxidase, p91^phox^ (Hs00166163_m1), p22^phox^ (Hs00164370_m1), p40^phox^ (Hs00241129_m1), p47^phox^ (Hs00165362_m1), p67^phox^ (Hs01084940_m1) and GAPDH (Hs99999905_m1) were purchased from Thermo Fisher Scientific.

### DNA transfection of Macrophages

DNA transfection of macrophages was performed using 4D-Nucleofector system (Lonza) with the P3 Primary Cell Kit (V4SP-3096) (Lonza). Macrophages (6 × 10^5^ cells) were mixed with 2 μg pCMVp47^phox^ or empty vector in 100 μL of P3 primary cell solution. Cells were transfected using the 4D-Nucleofector program DP-148. After transfection, cells were cultured in D-10 for 7 h at 37 °C and then assayed for ROS as described above. The expression of p47^phox^ was confirmed by western blot analysis.

### Silencing of p47^phox^ or TAK-1 in Macrophages by siRNA

Macrophages were transfected with p47^phox^ specific siRNA (#4392420, Thermo Fisher Scientific) or scrambled control siRNA (SR30004, Origene) using Lipofectamine 2000 (Thermo Fisher Scientific)^32^. Briefly, MDMs (0.6 × 10^6^/well) were seeded in a 24-well plate. 50 pmol siRNA and 3 μL Lipofectamine 2000 were mixed in 350 μL plain DMEM. The transfection mixture was incubated at room temperature for 30 min. Macrophages were washed with plain DMEM once and then incubated with the transfection mixture at 37 °C. After 2 h, the transfection mixture was removed and replaced with D-10 media. The level of p47^phox^ knockdown was assessed by RT-PCR and Western blotting 48 h post transfection. TAK-1 specific siRNA (#4390824, Thermo Fisher Scientific) was transfected into macrophages using the 4D-Nucleofector system with the P3 primary cell kit (Lonza)^32^. M-Mac was transfected with 100 pmol of siRNA following a transfection protocol by the vendor, and then cultured for 48 h. The cells were treated with or without 100 ng/ml of IL-27 for another 48 h and then subjected to western blot for the detection of TAK-1 protein or ROS assay as described above.

### SDS-PAGE and Western blot analysis

Macrophages or iDCs (1.5 × 10^6^ cells) were seeded in 6-well plates and cultured in 3 mL of D-10 or G4 media at 37 °C. To obtain whole-cell extracts for SDS-PAGE, cells were washed with ice-cold PBS, and resuspended in RIPA buffer (Boston Bioproduct) with protease inhibitor cocktail (Sigma Aldrich) and phosphatase inhibitors (Thermo Fisher Scientific) at 4 °C for 10 min. The protein concentration was determined using the bicinchoninic acid (BCA) protein assay kit (Thermo Fisher Scientific). Using a total of 25 μg protein, Western blot analysis was performed as previously described^32^. Antibody binding was visualized using the ECL Prime Western Detection Reagent (GE-Healthcare) and LAS-4000 (Fujifilm, Tokyo, Japan). The intensity of the band was analyzed by NIH Image J (http://rsbweb.nih.gov/ij/).

### 2D-PAGE and Western blot analysis

Whole cell lysate protein (100 μg) were subjected for 2D-PAGE analysis. For the first dimension of 2D-PAGE, immobilized pH gradient gel strips (pH 6–11, GE Healthcare Bioscience) were used. The samples were mixed with 100 uL of sample buffer and rehydrated for 18 h. The electrophoresis voltage was increased stepwise from 0 to 5,000 or 8,000 V for 3–5 h as recommended by the supplier. Each strip was equilibrated in 50 mM Tris-HCl (pH 8.8) containing 6 M urea, 2% SDS, 30% glycerol, and 20 mM DTE for 20 min. The second-dimension separation was achieved by performing SDS-PAGE in the manner described above. After separation by 2D-PAGE, the samples were transferred to a membrane for western blot analysis.

### De-phosphorylation of cell lysate

A total cell lysate (100 μg) from PMA-stimulated I-Mac were treated with 4000 units/ml of Lambda Protein Phosphatase (New England Biolabs) at 37 °C for 60 min in Lambda phosphatase buffer (New England Biolabs) supplemented with protease inhibitor cocktail, and then the lysate was subjected to 2D-PAGE followed by Western blot analysis.

### Statistical Analysis

Statistical analyses were performed using GraphPad Prism 5 software. Error bars indicate standard deviations (SD) or standard errors (SE) from means as noted. An unpaired Student’s t test was used and p values lower than 0.05 were considered significance.

## Additional Information

**How to cite this article:** Sowrirajan, B. *et al*. Interleukin-27 Enhances the Potential of Reactive Oxygen Species Generation from Monocyte-derived Macrophages and Dendritic cells by Induction of p47^phox^. *Sci. Rep.* **7**, 43441; doi: 10.1038/srep43441 (2017).

**Publisher's note:** Springer Nature remains neutral with regard to jurisdictional claims in published maps and institutional affiliations.

## Supplementary Material

Supplementary Dataset
