# Supplementary material for: Interleukin-27 Enhances the Potential of Reactive Oxygen Species Generation from Monocyte-derived Macrophages and Dendritic cells by Induction of p47phox
Source: Sci Rep. 2017 Feb 27;7:43441. doi: 10.1038/srep43441 (PMC5327488; doi:10.1038/srep43441)
Supplement: Supplementary Dataset [file srep43441-s1.pdf]

Supplementary Figures

Interleukin-27 Enhances the Potential of Reactive Oxygen Species Generation from Monocyte-derived Macrophages and Dendritic cells by Induction of p47^{phox}

Bharatwaj Sowrirajan, Yoshiro Saito, Deepak Poudyal, Qian Chen, Hongyan Sui, Suk See DeRavin, Hiromi Imamichi, Toyotaka Sato, Douglas B. Kuhns, Noriko Noguchi, Harry L. Malech, H. Clifford Lane and Tomozumi Imamichi.

Supplementary Figure S1

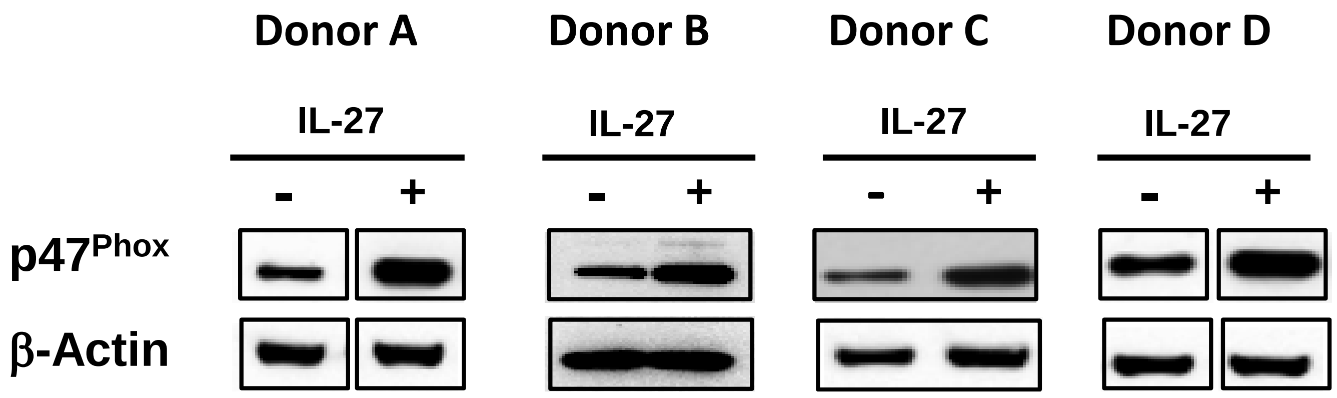

Supplementary Figure 1. IL-27 induces p47^{phox} protein. Monocytes from four independent healthy donors (A~D) were differentiated to macrophages (M-Mac) with M-CSF as described in the Methods section. The differentiated macrophages were treated with 0 or 100 ng/ml of IL-27 for 48 h and then whole cell lysates were prepared using RIPA buffer. Western blot analyses were performed using anti-p47^{phox} and anti-β-actin antibodies. The intensity of the band was analyzed by NIH Image J and normalized p47^{phox} intensity with β-Actin.

Supplementary Figure S2

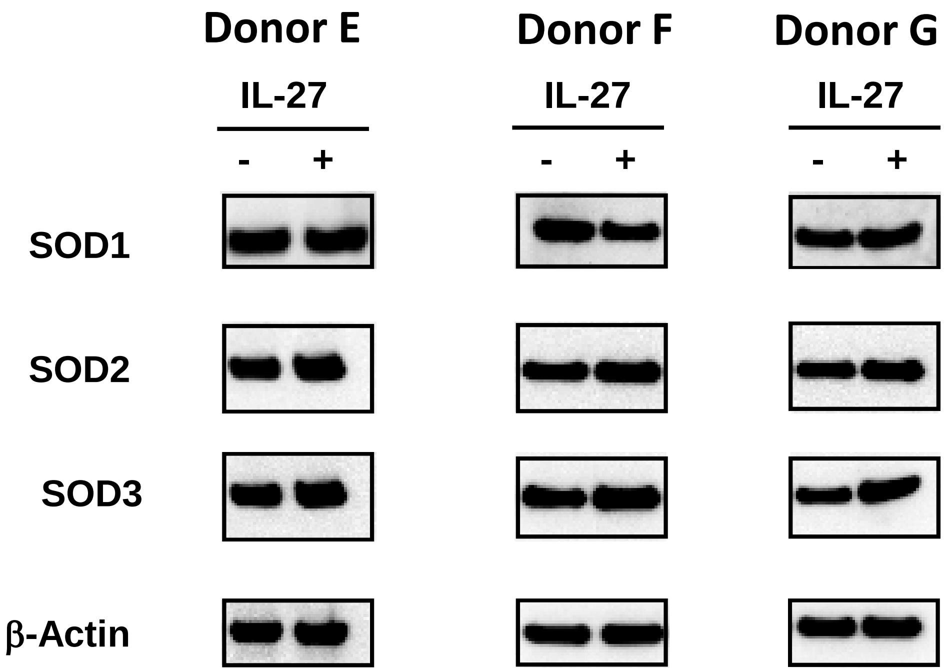

Supplementary Figure S2. **IL-27 has no impact on SODs induction.** Monocytes from three independent healthy donors (E~G) were differentiated to macrophages (M-Mac) with M-CSF as described in the Methods section. The differentiated macrophages were treated with 0 or 100 ng/ml of IL-27 for 48 h and then whole cell lysates were prepared using RIPA buffer. Western blot analyses were performed using anti-SOD1, anti-SOD2 and anti-SOD3 and anti-β-actin antibodies.

Supplementary Figure S3

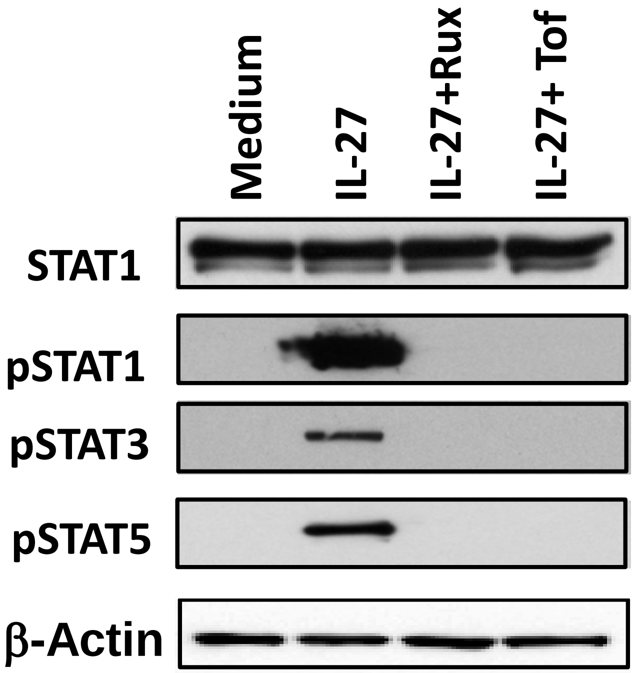

Supplementary Figure 3. JAK1/2 and JAK3 inhibitor suppresses IL-27-mediated STAT activation. M-Mac were treated with 1 μM of Ruxolitinib (Rux) or 5 μM of Tofacitinib (Tof) in D-10 for 1 h at 37 °C, and then cells were stimulated with 0 or 100 ng/ml of IL-27 for 5 min. Whole cell lysates were prepared as described in the materials and methods, and then western blot was carried out using anti-phosphorylated-STAT1, 3 and 5 antibodies, total STAT-1 and b-actin were detected by using anti-STTA1 and anti-b-Actin, respectively, for detecting in load control.

Supplementary Figure S4

Figure S4a

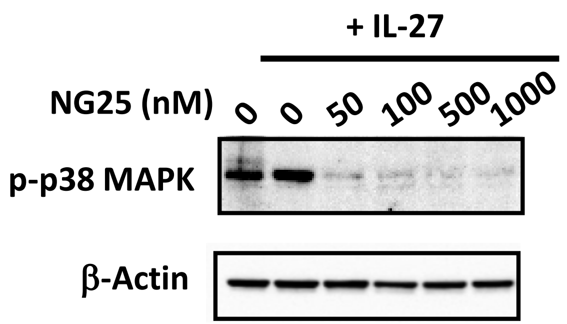

Figure S4b

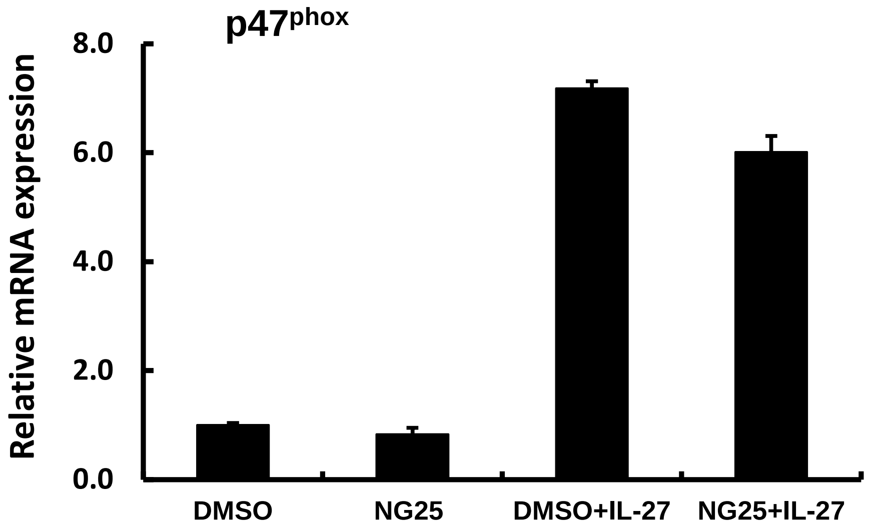

Figure S4c

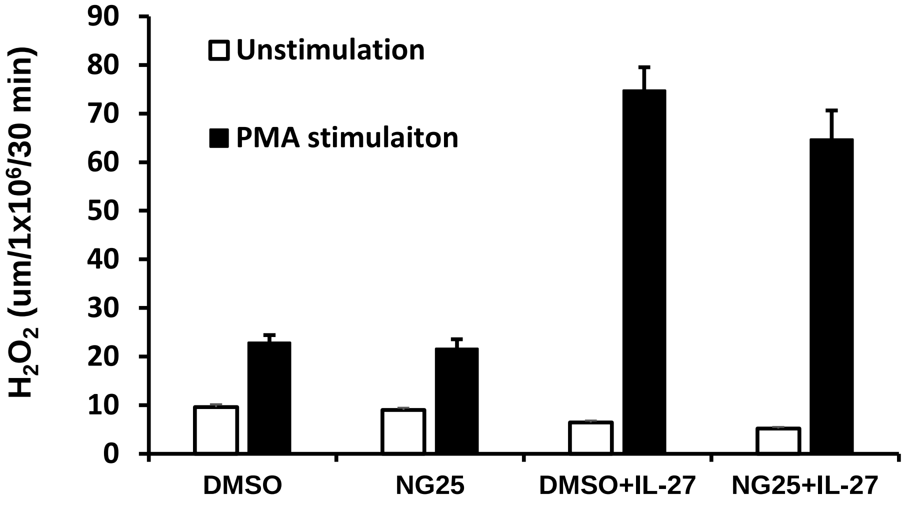

Supplementary Figure 4. TAK-1 inhibitor, NG25 had no impact on p47phox expression and ROS generation. (a) M-Mac were treated with different concentration of NG25 for 60 min, and then stimulated with 100 ng/ml of IL-27 for 10 min. Activation of p38 MAPK was detected by Western blotting using anti-phosphorylated p38 MAPK. (b and c) M-Mac were treated with 100 nM NG25 for 60 min and then incubated with IL-27 for 48 h. (b) p47^{phox} expression and (c) PMA-induced ROS generation were measured as described in the experimental procedures. Data shown represent means $\pm$ SDs of triplicate samples from two independent experiments.

Supplementary Figure S5

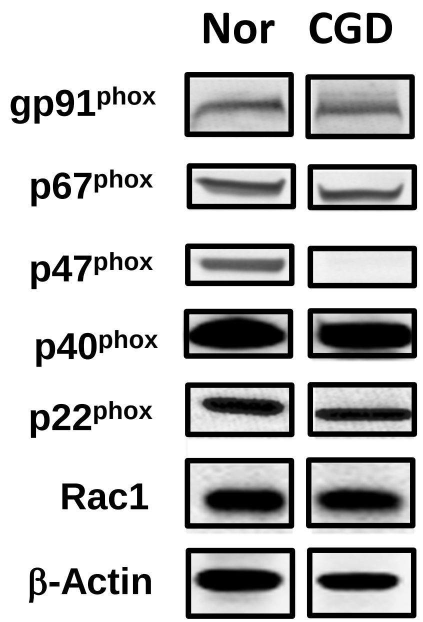

Supplementary Figure 5. **CDG$^{47-/47-}$ PBMC does not expresses p47^{phox}.**

Whole cell lysates of PBMC from a normal donor or a CGD$^{p47-/p47-}$ were prepared using RIPA buffer as described in the materials and methods, and then western blot was carried out using anti-gp91^{phox}, anti-p67^{phox}, anti-p47^{phox}, anti-p40^{phox}, anti-p22^{phox}, Rac1 and β-actin antibodies. β-actin was used as a loading control.

Supplementary Figure S6

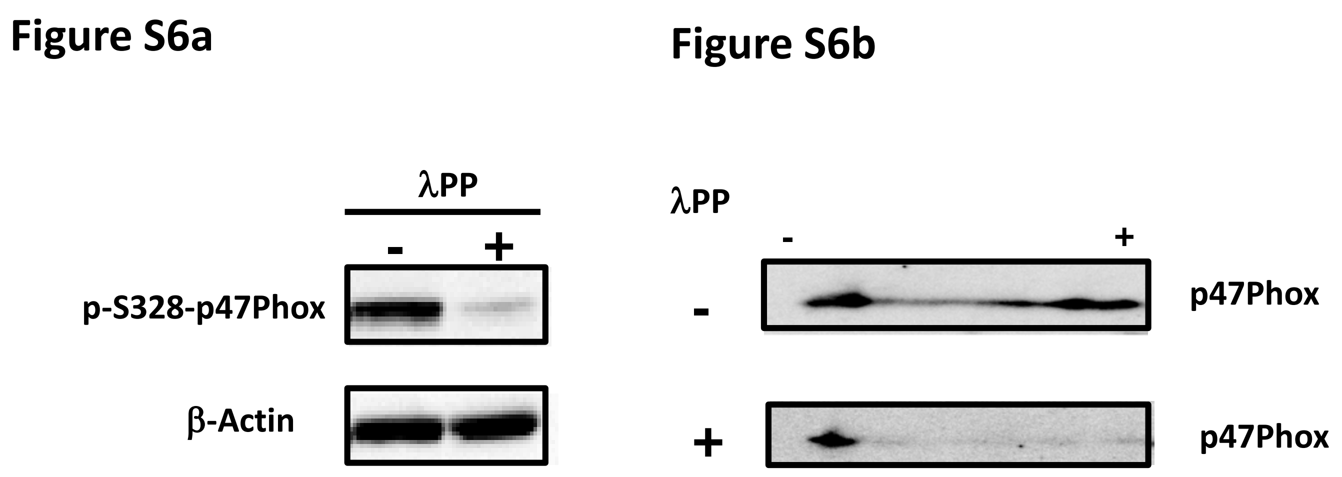

Supplementary Figure 6. Phosphatase treatment suppresses a shift of p47^{phox} bands to (+) side. IL-27 induced macrophages (I–Mac) was stimulated with PMA for 15 min and then whole cell lysate were prepared using RIPA lysis buffer in the presence of proteinase and phosphatase inhibitors as described in the Methods section. The lysate (100ug) was then treated with or without Lambda protein phosphatase (λPP). The lysates were separated on (a) 1 D- or (b) 2D-gels, and then Western blot analysis was performed using (a) anti-phosphorylated S328 p47^{phox} or (b) anti-p47^{phox} antibodies. The phosphatase treatment diminished the phosphorylation bands by 86 % in the 1D gel (a), and suppressed the shift of total of p47^{phox} bands to (+) side in a 2D gel. The intensity of the band was analyzed by NIH Image J.

Supplementary Figure S7

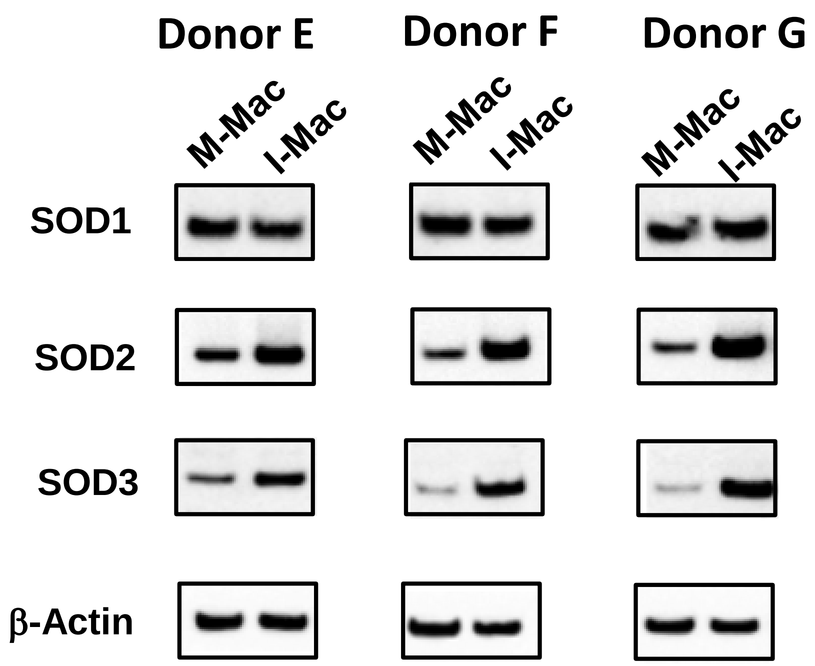

Supplementary Figure S7. **Expression of SOD2 and SOD3 in I-Mac are increased compared to M-Mac.** Monocytes from three independent healthy donors E~G were differentiated to M-Mac with M-CSF and I-Mac with M-CSF and IL-27 as described in the Methods section. Whole cell lysates of M- and I-Mac were prepared using RIPA buffer. Western blot analyses were performed using anti-SOD1, SOD2 and SOD3 and anti-β-actin antibodies. The intensity of the band was analyzed by NIH Image J and normalized each SOD intensity with β-Actin.

Supplementary Figure S8

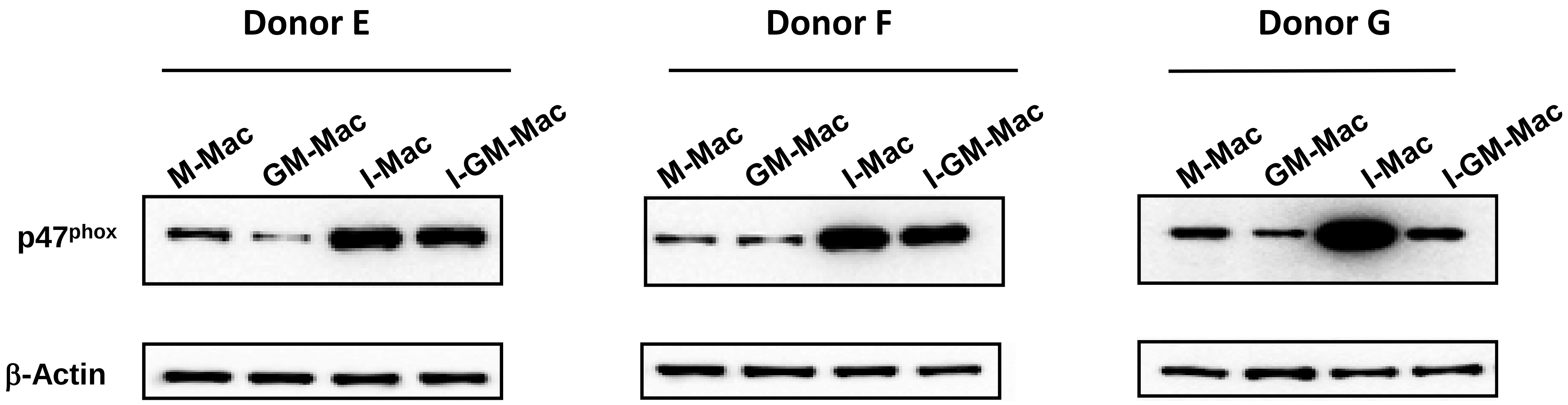

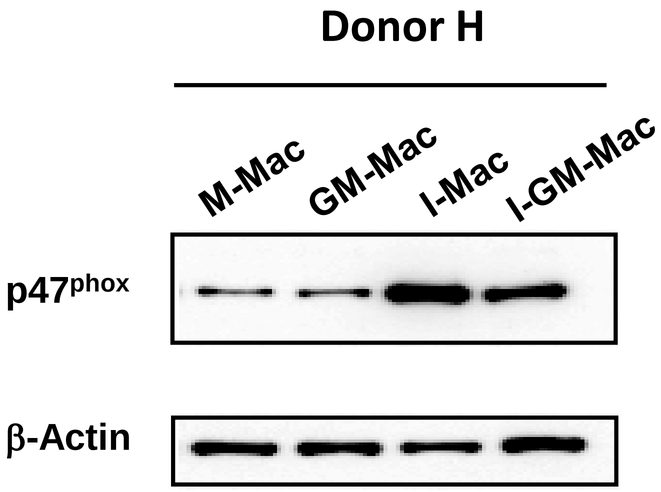

Supplementary Figure S8. **Expression of p47phox are induced in IL-27-induced GM-Mac.** Monocytes from four independent healthy donors E~H were differentiated into M-Mac, GM-Mac, I-Mac as described in the Method section. I-GM-Mac (IL-27-induced GM-Mac) were generated from the CD14+ monocytes by culturing for 7 days in the presence of 50 ng/ml of GM-CSF with 100 ng/ml of IL-27. Whole cell lysates of each cells were prepared using RIPA buffer. Western blot analyses were performed using anti-p47^{phox} , and anti-β-actin antibodies. The intensity of the band was analyzed by NIH Image J and normalized p47^{phox} intensity with β-Actin.

Supplementary Figure S9

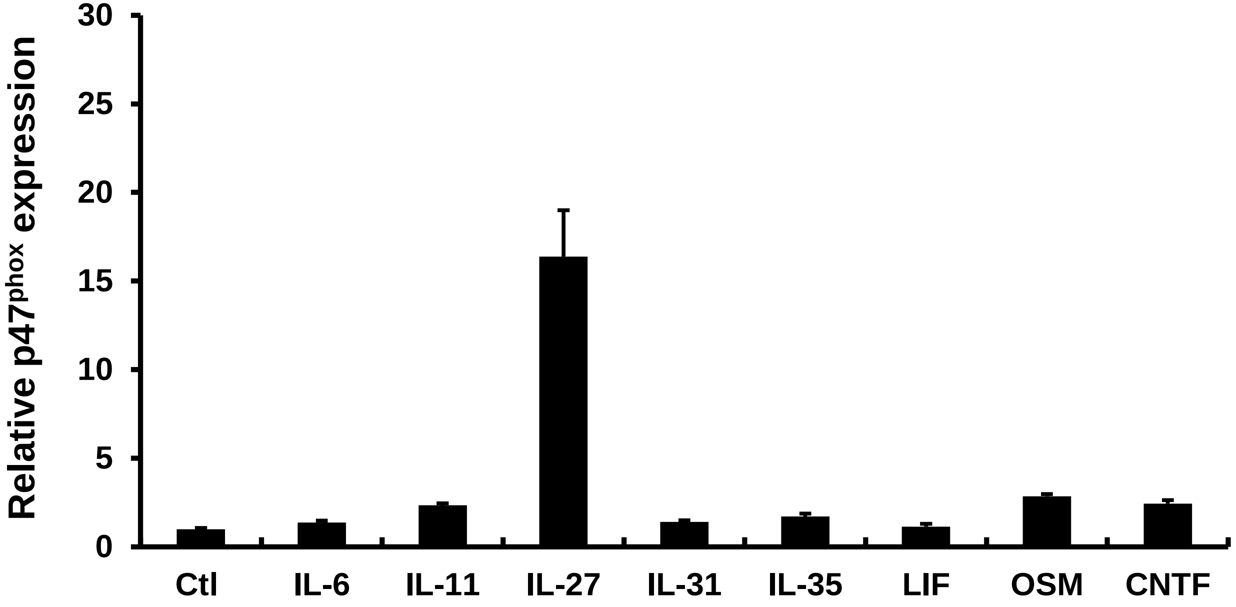

Supplementary Figure S9. Only IL-27 in the family cytokine enhances p47^{phox} expression. M-mac was incubated with 100 ng/ml of IL-6, IL-11, IL-27, IL-31, IL-35, LIF, OSM or CNTF for 48 h and then p47^{phox} expression was determined by real-time RT-PCR. Data shown represent means ± SDs of triplicate samples from two independent experiments.
